# ‘It’s quite difficult to put Autistic relationships in a box’: A qualitative exploration of romantic relationships in gender and sexually diverse Autistic adults

**DOI:** 10.1177/13623613251407765

**Published:** 2025-12-29

**Authors:** Tina Ciric, Luka CJ White, Claire Allison-Duncan, Ellen Maloney, Karri Gillespie-Smith

**Affiliations:** 1 The University of Edinburgh, UK

**Keywords:** autism, gender minority, relationships, sexual minority

## Abstract

Autistic people enjoy friendships and sexual and romantic relationships. Many Autistic people are also part of a gender or sexual minority, yet gender and sexual minorities are often under-represented in autism research. The current study asked, ‘How do gender and sexually diverse Autistic adults experience and perceive romantic relationships?’ Sixteen gender and/or sexual minority Autistic adults took part in semi-structured interviews. Interviews were transcribed and analysed using thematic analysis. Four main themes were identified from the interview data: (1) ‘It’s Quite Difficult to Put Autistic Relationships Into a Box’, (2) Challenging Social Norms, (3) The Perks and Perils of Online Dating and (4) Understanding and Neurotype. Overall, participants described their relationships as individual, inherently valuable and shaped by unique preferences, mutual understanding and explicit communication. They also emphasized the fluidity between friendships and romantic partnerships, the importance of mutual understanding and the inherent value of rejecting prescriptive social norms in favour of individualized romantic relationship dynamics. These findings highlight relationship structures in Autistic groups who identify as gender and/or sexual minority.

**Lay abstract:**

Autistic people enjoy friendships and sexual and romantic relationships. A proportion of the Autistic community identify as being a part of a gender and sexual minority (e.g., trans, non-binary, gay, lesbian, etc.), yet this group is often under-represented in autism research. The current study focused on this group specifically and asked them, ‘How do gender and sexually diverse Autistic adults experience and perceive romantic relationships?’ Sixteen gender and/or sexual minority Autistic adults took part in interviews. Interviews were transcribed and analysed using thematic analysis. Four main themes were identified from the interview data: (1) ‘It’s Quite Difficult to Put Autistic Relationships Into a Box’, (2) Challenging Social Norms, (3) The Perks and Perils of Online Dating and (4) Understanding and Neurotype. The participants described their relationships as individual, valuable and shaped by unique preferences, mutual understanding and clear communication. They also talked about how the lines between friendships and romantic partnerships can be blurred, for example, sometimes they have sex with their friends and don’t see this as an activity exclusively for romantic relationships. They also mentioned the importance of mutual understanding and the inherent value of rejecting social norms in favour of more individualized romantic relationships.

Research focusing on Autistic^1^ people’s relationships and sexuality has either included those who are part of gender and sexual minorities (GSMs) in larger heterosexual cisgender groups (e.g., Barnett & Maticka-Tyndale, 2015; Sala et al., 2020) or not included them at all (e.g., Cheak-Zamora et al., 2019; Smith et al., 2021). To date, there has been no research focusing on Autistic GSM relationships specifically, which is important given that additional intersectionality will influence relational experiences (Jackson-Perry, 2024).

Empirical projects in Autistic GSM groups have explored mental health issues with some research reporting Autistic SM adults show poorer mental health and lower quality of life compared to Autistic heterosexual adults (McQuaid et al., 2023). More recently, research looked at mechanisms contributing to or preventing poor mental health outcomes in Autistic GSM adults showed that increased minority stress and camouflaging rates (common in groups who are multiply marginalized) lead to higher levels of poor mental health outcomes (White et al., 2024). Understanding how GSM Autistic adults experience and perceive romantic relationships is therefore crucial, given the role of romantic relationships in preventing or contributing to poor mental health outcomes (Braithwaite & Holt-Lunstad, 2017; Mirsu-Paun & Oliver, 2017). The current study therefore aims to address this knowledge gap by exploring the experiences and perceptions of romantic relationships in Autistic GSM groups.

There is a lot of theoretical and discussive literature exploring GSMs in Autistic groups; however, there remains a dearth of empirical research (Strang & Fischbach, 2023). The limited empirical research that has been carried out has focused on a mixture of topics including qualitative experiences of gender non-conformity and/or gender dysphoria (Coleman-Smith et al., 2020; Cooper et al., 2020; Kourti & MacLeod, 2019; Oliver et al., 2025), as well as experimental studies on gender-related cognition that reported how Autistic transgender people show implicit gender biases for their affirmed gender and not sex assigned at birth (Kallitsounaki & Williams, 2022).

Despite this progress, little is known about how Autistic GSM adults define and navigate *romantic* relationships. Due to this gap, it is difficult to measure relationship frequency and quality since Autistic experiences of relationship type and quality differ from neurotypical perceptions, with more asexuality reported in Autistic romantic relationships and reduced emphasis on sex-determining romantic classification (Sala et al., 2024). Studies that have included GSM Autistic people in their sample note similar challenges, such as difficulties understanding neuronormative social cues, using ‘appropriate’ dating scripts (which are often informed by neurotypical expectations), barriers to meeting partners and communicating intimacy needs (Lewis et al., 2021; Sala et al., 2020).

Building on this literature, the present study centres the voices of Autistic GSM adults to address the question:

*How do gender and sexually diverse Autistic adults experience and perceive romantic relationships?*

## Method

### Design

#### Methodological approach

The current study employed a hermeneutic phenomenological approach: phenomenology being the focus on understanding lived experiences, and hermeneutics being the interpretation of their essence (van Manen, 1990). It is also important to note that the analytic process adopted queer theory and constructivist philosophical approaches, which acknowledge sexuality and gender as socially constructed phenomena (Spencer et al., 2014). Therefore, the narratives shared by participants regarding their experiences and understanding of romantic relationships are not considered exclusive products of the individual but rather reflections of their interactions within their broader social environment. Interviews were semi-structured to allow for a broad coverage of relevant topics while also granting the flexibility to tailor interviews to each participant and probe further as necessary. An inductive, data-driven approach to thematic analysis was used to ensure the most flexible comprehension of the data, avoiding any attempts to force it into pre-existing theories or coding frameworks.

### Participants

Participants consisted of 16 Autistic adults (see Table 1 for demographic information), who met the following eligibility criteria: (1) aged over 16, (2) English-speaking and able to communicate verbally or through writing, (3) located within the United Kingdom, (4) self-diagnosed or medically diagnosed as Autistic, (5) gender identity not fitting into the gender binary or not aligned with their assigned sex at birth and/or (6) sexual minority identification. A self-diagnosis of autism was considered sufficient since this approach is proposed to be more autism-affirming and critical in the depathologization of autism (Overton et al., 2023). Pansexuality was deemed sufficient for inclusion regardless of gender identity due to its inherent rejection of the gender binary (Rice, 2015). Participant ages ranged from 20 to 46 years (*M* = 27.90, *SD* = 8.58), with the majority (*n* = 13) ethnically identifying as White British or Irish.

**Table 1.** Participant demographics.

Participant	Age	Gender	Pronouns	Sexual orientation	Age and method of autism diagnosis	Other diagnoses	Ethnicity
Eli	20	Transmasculine	He/They	Bisexual	SD, 18	None	White (Polish)
Sidney	20	Neutrois	He/Him	Bisexual, Polyamorous	SD, 17	MHC, PHC	White (British, Irish)
Amara	23	Non-binary	She/Her	Lesbian, Queer	MD, 23	LD, PHC	White (Other)
Sam	22	Non-binary	He/They	Bisexual, Queer	MD, 20	LD, PHC, NDD	White (British Jewish)
Jonathan	21	Non-binary	Any	Attracted to masculinity	MD, 21	MHC, PHC, NDD	White (British, Irish)
Iris	28	Non-binary	She/They	Bisexual, Pansexual	MD, 10	PHC	White (British, Irish)
Darren	25	Transgender man	He/Him	Gay	SD, 20	LD	White (British, Irish)
Masie	27	Transgender woman	She/Her	Pansexual	SD, 25	MHC, seeking NDD	White (British, Irish)
Renee	22	Non-binary	They/Them	Lesbian	MD, 20	MHC	White (British, Irish)
Zia	43	Non-binary	They/Them	Bisexual	MD, 40	MHC, PHC, NDD	Other
Marcy	25	Gender fluid	She/They	Pansexual	SD, 24	MHC	White (British, Irish)
Sarah	33	Gender fluid	Any	Bisexual	MD, 33	MHC, NDD	White (American)
Alana	46	Agender	She/They	Asexual	MD, 39	MHC, PHC, NDD	White (British, Irish)
Melinda	30	Non-binary	They/Them	Pansexual	MD, 27	PHC	Other
Cleo	20	Gender fluid	They/Them	Pansexual	MD, 17	LD, MHC	White (British, Irish)
Penny	41	Cis woman	She/Her	Pansexual	SD, 40	NDD	White (British, Irish)

Note. All collected participant demographic information (*N* = 16); all names are anonymized. SD: self-diagnosed; MD: medically diagnosed; MHC: mental health condition; PHC: physical health condition; LD: learning disability; NDD: other neurodivergent diagnosis.

### Procedure

Following ethical approval from the Ethics Committee in Clinical and Health Psychology at the University of Edinburgh (No. CLIN821), participants were recruited through online platforms including Twitter, Instagram, Facebook, Reddit and Discord. A social media ad was used to advertise the study, and those interested clicked the QR code that led to a Qualtrics survey containing the information sheet and consent. Once participants gave their consent, the survey collected their demographic, contact, emergency contact and diagnosis details, as well as their preferred method of interview. If they indicated they did not wish to participate, they were instead guided to the debrief document.

All interviews were conducted remotely, and participants were given the option of using (1) Microsoft Teams video call (with the camera on or off according to personal preference) (2) telephone or (3) Microsoft Teams chatbox. Two researchers were responsible for the interviews: one of whom was neurotypical, and the other neurodivergent. Interview questions were provided in advance, and interview times ranged from 29 to 95 min (*M* = 44 min; *SD* = 18 min). Two interviews were exclusively text-based. At the end of each interview, participants were also given the chance to further elaborate on past topics or bring up anything else that they felt was important or relevant. Participants chose their own pseudonym; however, if there were duplicate pseudonyms, these were replaced by names assigned by the researcher.

### Analysis

**Figure 1.**
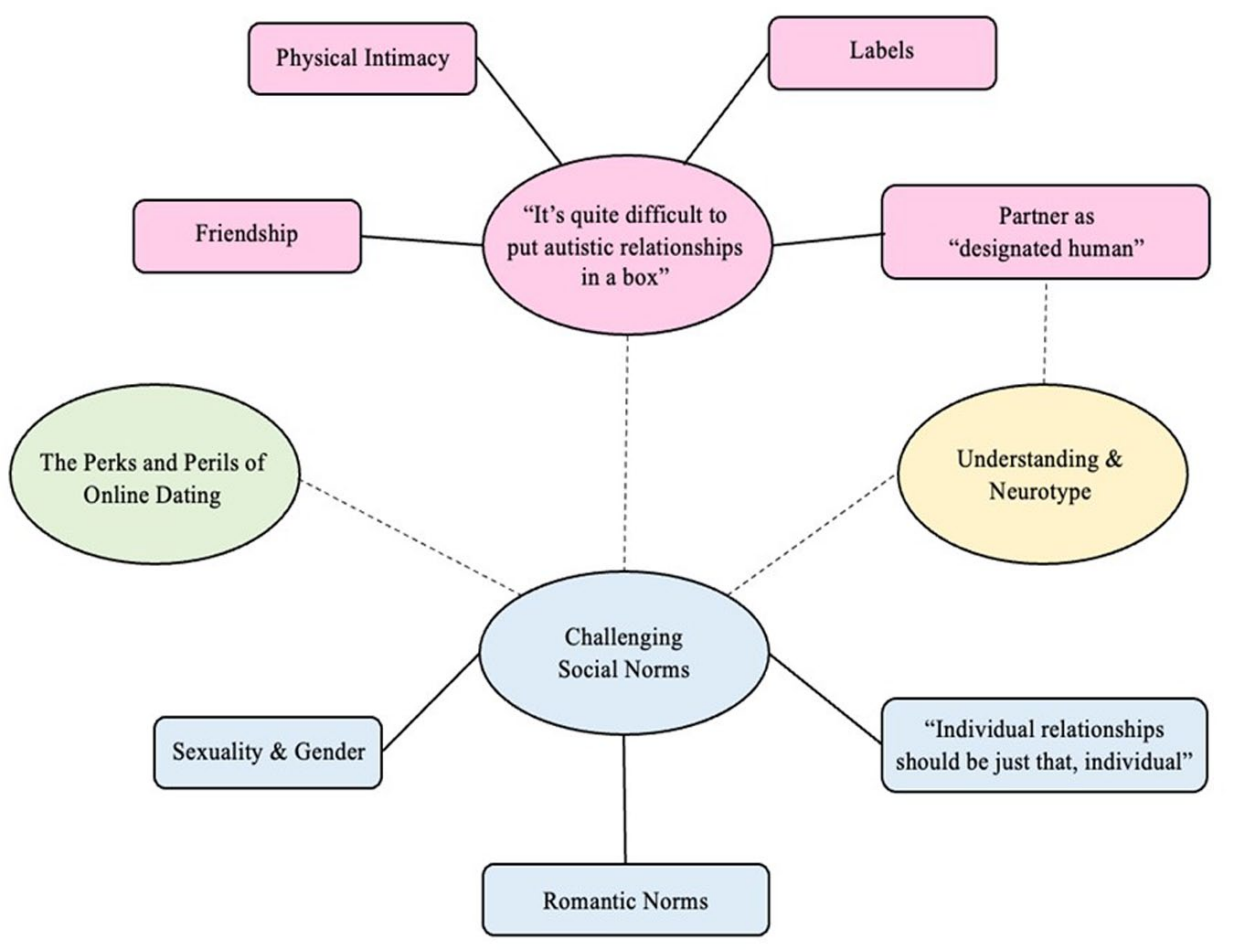
Final thematic map. Note. The four ovals represent themes, while the rectangles attached with a solid line represent subthemes. A dotted line indicates a theoretical connection between themes.

Data analysis followed Braun and Clarke’s (2022) six-phase approach to thematic analysis. Interview transcribing was shared across three researchers, after which the interviewer checked each transcript to ensure accuracy. Two researchers were subsequently involved in the analysis: one of whom is cisgender and neurotypical, one of whom is transgender and is Autistic. Initial coding was done manually by one researcher, and then each researcher generated their own mind-maps of codes to facilitate visualization of the commonalities across the data set. Each researcher then generated their initial thematic map, and these were then discussed and taken into consideration to construct the final thematic map (Figure 1). Finally, salient data excerpts across each theme and subtheme were chosen, and an analytic narrative was written.

### Reflexivity statement

Hermeneutic phenomenology recognizes that individuals construct and interpret the meaning of their experiences based on their cultural, social and historical contexts (van Manen, 1990). This also applies to researchers themselves, as they bring their own biases, assumptions and interpretations to the analytic process. The current study was conducted by a diverse research team comprising both Autistic and neurotypical researchers, as well as both transgender and cisgender researchers, ranging from undergraduate to senior researcher levels. The analysis involved collaboration between a neurotypical cisgender researcher and an Autistic transgender researcher. This diversity within the team allowed for the integration of an Autistic lens during analysis and reflexive discussions, ensuring a balanced interpretation of the data.

## Results

Four main themes were identified from the interview data: (1) ‘It’s Quite Difficult to Put Autistic Relationships Into a Box’, which contains four subthemes, (2) Challenging Social Norms, which contains three subthemes, (3) The Perks and Perils of Online Dating and (4) Understanding and Neurotype (see Table 2). It is important to note that the quotations chosen are representative of the sample and the themes.

**Table 2.** Overview of themes, subthemes and example quotes.

Themes	Subthemes	Example quotes
1. ‘It’s quite difficult to put Autistic relationships into a box’ *This theme encompasses the deeply individual and fluid nature of Autistic romantic relationships, where traditional boundaries between friendships and romantic partnerships are often blurred.*	1.1 Friendship *This subtheme captures how friendships often form the foundation of romantic relationships, with participants emphasizing shared understanding, trust and emotional connection.* 1.2 Physical intimacy *This subtheme explores diverse perspectives on the role of physical or sexual intimacy in defining romantic relationships.* 1.3 Partner as ‘designated human’ *This subtheme highlights the unique centrality of romantic partners in participants’ lives, emphasizing safety, emotional closeness and integration into daily life.* 1.4 Labels *This subtheme reflects the importance of mutual agreement in defining relationships as romantic, with labels playing a more significant role than specific behaviours.*	Nova: ‘I think like cause the thing is, my partner is basically my best friend’. Eli: ‘Autism is such a wide spectrum that relationships are bound to have differences and similarities, and where I might not find physical closeness particularly important or necessary, I know other autistic for whom that is the most important aspect’. Amara: ‘I think it’s just how our lives are like connected. Whereas my friends and I, we live our own lives and we intersect in our lives. But, like my partner and I, our lives they’re connected in a very different way than that’ Sidney: ‘I guess the only thing I would say that really like cements a romantic relationship, as we’re dating and we’re together and we’re partners, would be a label’.
2. Challenging Social Norms *This theme examines the challenges participants face in navigating societal expectations around romantic relationships, as well as their ability to redefine these norms to suit their individual needs.*	2.1 Navigating neurotypical romantic norms *This subtheme addresses difficulties understanding and adhering to traditional dating and relationship scripts, which often lead participants to question or reject them.* 2.2 ‘Individual relationships should be just that, individual’ *This subtheme focuses on participants’ rejection of prescriptive societal norms, emphasizing explicit communication and customized relationship dynamics.* 2.3 Sexuality and gender *This subtheme explores how participants’ identities as gender and sexual minorities further influence their rejection of normative relationship frameworks, fostering alternative and individualized approaches.*	Eli: ‘Rules that suggest a partner can’t flirt with their friends, can’t like certain people’s posts, or can’t dress a certain way (which I have seen more often in non-autistic relationships) have always baffled me somewhat’. Masie: ‘Hey, if you find it hard to anticipate the kind of standardized expectations that come with relationships, they’re not intuitive to you. You should try relationship anarchy because it’s all explicit. And then it’s easier and it kind of, yeah, it’s-it’s good that way. Uh, so I think that’s a thing, which is kind of useful, can be useful for autistic people when they date’. Maisie: ‘And so you can afford to be a massive slut, you know and it’s kind of like because you weren’t ever gonna be able to win that game anyway. So you’re like, not even playing. And it’s the same reason that like a lot of gay people are kind of a bit weirder in terms of how they build their relationships’.
3. The Perks and Perils of Online Dating *This theme explores participants’ experiences with online dating, highlighting its ability to increase access to diverse potential partners while also presenting challenges in navigating the unspoken rules and scripts of virtual spaces.*		Darren: ‘Online stuff is a whole other new set of social skills. And, um, I did enough work trying to get the in-person set, I think I’m a bit tired of trying to learn an entirely new one’.
4. Understanding and neurotype *This theme centres on the importance of mutual understanding in romantic relationships, with participants highlighting the ease of communication and shared experience in Autistic partnerships. It also emphasizes the effort required to build understanding in relationships between Autistic and non-Autistic partners.*		Darren: ‘So, one of my previous partners was also autistic . . . And I found that like . . . consideration, and . . . communication, were kind of the main things that were different. In the sense that where . . . in that relationship I found that the other person was a lot more . . . considerate of me, and my kind of . . . what would you call them, quirks, and things that might be stressful or difficult’.

### Theme 1: ‘it’s quite difficult to put Autistic relationships into a box’

This theme encompasses the difficulty in categorizing Autistic relationships due to the significance of traditional factors such as physical intimacy and exclusivity varying across individuals. Eli noted ‘it’s quite difficult to put Autistic relationships into a box’ which refers to the diverse ways in which Autistic people have relations with others and how sex does not necessarily indicate a romantic relationship. There were nuanced and interconnected understandings of relationships, with fluid boundaries between friendships and romantic partnerships. This theme reflects the personalized nature of relationships and challenges traditional conceptualizations of romantic relationships. Four subthemes were identified: *Friendship, Physical Intimacy, Partner as ‘designated human’* and *Labels*.

#### Subtheme 1.1: friendship

Overall, participants described friends as highly valued people, with whom there was shared understanding, trust and feelings of safety. When asked to describe the distinction between friends and romantic partners, participants noted similar notions of connection across both types of relationship:

Masie: ‘I value my friends as much as I value my partners, and I feel like those things are very similar’.

Many participants referred to their partner as their ‘best friend’, suggesting friendship as a necessary foundation for a romantic relationship. However, the exact distinction between a romantic partner and a best friend remained generally unclear:

Masie: ‘So like when I’m close to a friend, I like build a very rich connection with them. And with partners, it’s similar thing I build a very sort of rich connection with them. I’d say I find the kind of distinction between like romantic and non-romantic connections to be kind of hard to understand’.

#### Subtheme 1.2: physical intimacy

Participants expressed a range of perspectives on whether physical or sexual intimacy served as a boundary between friendships and romantic relationships. While some viewed sex as something that was reserved for a romantic partner, others felt it was more fluid or questioned its overall significance in defining relationship boundaries:

Sidney: ‘I used to believe that I had to be in a relationship with everyone that I slept with. . ., so I would kind of jump from relationship to relationship. Have a very monogamous mindset of a romantic relationship is someone that you’re having sex with. But now I generally sleep with most of my friends, so that isn’t what makes a relationship for me anymore’.

Cleo: ‘It doesn’t have to be sexual, so I mean, so if you’re, like, not OK with that or I’m not OK, um or partner is not OK with that, and that’s totally fine. We don’t need to do anything at that, cause you can have a relationship without being sexual’.

Participants also acknowledged common stereotypes that Autistic people were not interested in sexual intimacy, or that it was less important to them because they were Autistic. This was widely seen as a harsh generalization:

Iris: ‘I do think it’s damaging when people assume that we are absolutely all disinterested in sex or incapable of experiencing sexual attraction or any or any of this other stuff because it fundamentally is a misunderstanding and it downplays things that are actually very important to some of us, you know’.

Regardless of the perceived role of sexual intimacy as a distinguishing factor between friends and partners, participants generally acknowledged the presence of a ‘different kind of feeling’ between friends and partners, which introduces our next subtheme.

#### Subtheme 1.3: partner as ‘designated human’

Participants generally agreed that a defining trait of romantic partners was the high level of companionship, understanding and safety to be themselves that they felt in their presence:

Melinda: ‘For me, it’s all about feeling safe in another person’s presence. Into being able to be yourself and drop any masks’.

Participants also described the high degree to which they integrated their lives with a romantic partner, with much higher levels of ‘closeness’ than one would have with other people.

Iris: ‘if you’re very invested, you may end up thinking about planning either growing old together. So yeah, I don’t think people do that with their friends with benefits usually’.

Romantic relationships were also described as a give-and-take, with partners having more obligations and looking out for one another to a higher degree, which could be difficult for some people:

Darren: ‘And also, I find romantic relationships can be very socially and emotionally demanding and difficult. In a way that friendships often aren’t, and they often come with difficult expectations’.

Participants also described a higher degree of emotional investment within romantic relationships, with partners having a large influence on their emotional state. This, paired with the high level of integration of lives between partners (such as the fusion of friend groups), meant that partners were very central figures in participants’ lives; Darren equated his partner to ‘gravity’, in that ‘things start to centre around this person’. This notion was also relevant within the context of breakups, as undoing this high level of integration was described as particularly distressing, with one participant describing a flare-up in suicidal thoughts following their breakup:

Amara: ‘So I think like losing both the person as a person, losing like the connections around that person, losing the routines that you’ve built up losing like, even just the stuff your physical environment changes. Like everything in your life is, it’s a big change’.

Ultimately, romantic partners were seen as uniquely central figures in participants’ lives, providing high levels of emotional intimacy and integration. As Marcy put it: ‘[It’s like] friendship, but with maybe some benefits as to this person is kind of stuck with you, this is your designated human’.

#### Subtheme 1.4: labels

While the defining characteristics of romantic relationships were considered to be fluid, participants generally echoed Darren’s statement that ‘if you basically get down to it it’s more about how you and the other person are defining the relationship rather than any actual listable differences’. For example, Sam made the point that the difference between getting coffee with a friend and getting coffee with a partner but calling it a ‘date’ was simply a difference in how it was framed:

Darren: ‘Obviously you’ve got the kind of, I’m going to call them “socially-understood factors”, such as levels of physical intimacy but I don’t think those inherently make something a relationship or a friendship in the sense where we generally consider holding hands, or kissing, or having sex with someone to be something that happens in a romantic relationship but also happens in friendships? But not all friendships. And that’s kind of that’s kind of decided by the people who are in those friendships’.

Sidney also described how with his last partner, their relationship was indistinguishable from when they called it a friendship to when they started framing it as a romantic relationship, stating that ‘the only thing that made it a romantic relationship, made us partners, was having a mutual desire to have a label’. Ultimately, it depends on whether both parties agree to define their relationship as a romantic partnership – or, as Iris aptly describes, both must ‘say the magic words’.

### Theme 2: challenging social norms

The theme of challenging social norms encompasses the complexities that Autistic GSM individuals face in navigating neurotypical societal expectations associated with romantic relationships. Many participants questioned the inherent value of the implicit rules, cues and scripts related to relationships. Building upon the previous theme of ‘It’s quite difficult to put Autistic relationships into a box’, participants advocated for defining connections based on individual preferences rather than adhering to prescriptive norms. Three subthemes arose: *Navigating Neurotypical Romantic Norms, ‘Individual relationships should be just that, individual’*, and *Sexuality and Gender.*

#### Subtheme 2.1: navigating neurotypical romantic norms

Participants often felt that they had a hard time understanding how they were supposed to navigate the neurotypical social scripts surrounding romantic relationships. In relation to dating in particular, participants felt like they were expected to ‘know the kind of patterns of interaction’, which Nova specifically describes as ‘just kind of a nightmare, it’s where I have to try to present myself in a way that I don’t necessarily know how to do’:

Nova: ‘I still don’t know what the dating rules are [laugh]. I don’t know when people say in shows wait two days to text. That like I don’t know whether that’s real or whether that’s, I do not know. So to this day, I still don’t understand what a typical dating relationship’s like, at least according to other people. And I think that’s a real crucial difference between Autistic and non-autistic people. We don’t know those things off the bat, there is a lot stacked against you’.

Difficulties in interpreting these norms and cues were also brought up as a factor that increased vulnerability to abuse. This was due to a tendency to believe how others presented themselves and take romantic interactions at ‘face value’, unknowingly putting themselves at risk. Alana states that for this reason ‘It’s very easy [for Autistic people] to not see past the charms of a predatory narcissist and to not know any better’:

Melinda: ‘I think there’s just almost a bit of inherit danger of like, particularly being an AFAB^2^ Autistic person and sort of navigating romantic relationships. Because I have for sure found that, taking people at face value put me in really shit situations because inherently being in romantic relationship is, that level of vulnerability and it just puts you in a really shit place if you happen to have fallen for someone who doesn’t have very good intentions’.

Ultimately, participants described that neurotypical social norms, cues and scripts surrounding romantic relationships made it more difficult for Autistic people to navigate such relationships:

Masie: ‘You kind of can’t participate in the sort of neurotypical like social landscape in the same way that neurotypical people can. You also aren’t invested in it in the same way’.

#### Subtheme 2.2: ‘individual relationships should be just that, individual’

It was widely agreed that ultimately it is up to each couple to decide what their rules and criteria are going to be for their individual relationship, regardless of societal norms. Whereas *Navigating Neurotypical Romantic Norms* looked at how romantic norms could be difficult to navigate for Autistic GSM participants, this subtheme explored how realizing that these norms are neurotypically orientated and informed allowed the participants to reject these expectations. They became empowered to move forward in their relationships governed by their own rules and expectations. This then allowed for individualization of what romantic relationships are for each participant; as noted by Sam, ‘you just make it up and then that’s fine’:

Penny: ‘a lot of social expectations around relationships are very prescriptive. Like I can remember being told by my friends that it was outrageous of me to not be able to define what cheating was without talking to my partner. And I was like, well, how do I? Surely cheating depends on what you and your partner agree is cheating. They’re like, no, it’s kissing anyone else. I’m like, well for some people. Yeah, so and that was when I was in my 20’s. So, I was already of that mindset of each individual relationship should be just that, individual’.

Something that multiple participants spoke about was the norm of monogamy, with Zia noting that their past relationships had ultimately failed because ‘they were both convinced they could talk me into having marriage and kids. It’s like, yeah, it’s not happening’. Participants were very vocal about not compromising on the things that were important to them in a relationship, and displayed strong boundaries:

Sidney: ‘Yeah, I do always make sure that before I get with someone they know fully, like my boundaries. And what I want and it’s up to them to know if they can handle that or not’.

At the end of the day, participants agreed that there was a much broader spectrum of what romantic relationships could look like than is generally portrayed. Rather than focusing on what relationships ‘should’ look like, participants emphasized the value of individualization based on the values and preferences of each person:

Iris: ‘my current primary partner is somebody who I am romantically in love with and he does not romantically love me back, but we are, for want of a better word, friends with benefits and adventure companions. And even though I’m well aware that there’s a mismatch in our feelings towards one another, I actually don’t particularly mind because he’s never failed to be an absolutely perfect adventure companion and absolutely incredible lover’

This reflects the flexibility and agency participants embraced in defining relationships on their own terms, and how in the end all that matters is that both parties are satisfied with the state of their relationship.

#### Subtheme 2.3: sexuality and gender

While this analysis has previously discussed understanding the neurotypicality of relationship norms as facilitating their rejection, participants also suggested that understanding the cisnormativity and heteronormativity of these norms could play a further role in this:

Sidney: ‘the trans/Autistic people I’ve dated just generally have a disillusionment with the cis/hetero institutions of marriage and children and stuff, so I don’t like know if it’s because we’re Autistic or if it’s because we’re trans’.

Darren: ‘I think this is possibly because I’m gay, a lot of things that some people think are exclusively to romantic relationships I don’t see as inherently being like that, so long as everyone is, like, on the same page about it, if that makes sense?’

Multiple participants also mentioned the high amount of overlap they had noticed between the autism and polyamory communities, as well as the high amounts of gender and sexual diversity within the Autistic community. In relation to the kink and polyamory communities in particular, the direct communication style and freedom granted by their inherent rejection of normative relationship frameworks was seen as appealing and beneficial:

Penny: ‘I think because of the freedom I have to communicate that exists within polyamory, reduction of expectations. Has definitely benefited me, especially where I am now because [I] probably wouldn’t be dating otherwise’.

Masie: ‘You know, the kink scene is great for Autistic people. You’ve maybe encountered this already in your research because there’s lots of explicit rules about how you’re supposed to interact with people. And that’s the space which is, like, very accessible’.

Therefore, the intersection of being an Autistic GSM and understanding the inherent neurotypicality, and cis- and hetero-normativity of traditional relationship norms, allowed for a greater diversity in relational dynamics.

### Theme 3: the perks and perils of online dating

In general, online dating was acknowledged as an appealing alternative to traditional avenues of meeting a partner, allowing participants to do so on their own terms:

Penny: ‘Absolute nightmare, because where do all the non-neurodivergent people meet? Clubs! Where do I hate with the passion of 1000 Suns? Clubs!’

Dating apps were described as ‘removing a lot of the obstacles’, ‘good for filtering people’, granting access to a ‘more diverse range of people’ and ‘speeding up the process’ of finding a partner. This was seen as particularly useful for GSMs when it came to finding potential partners or communicating their desired relationship structures (i.e., non-monogamy):

Jonathan: ‘another step, quite a massive step, when you’re gay, which is figuring out whether they’re queer in the first place. So, I think that Tinder and apps like that, they really speed along the process because you know they’re on there to date, in fact you know that everyone you match with is into you, so it’s even better’.

However, participants also noted certain difficulties associated with online dating, specifically surrounding neurotypical social norms, cues and scripts. Dating in a virtual space was described as the ‘neurotypical world on steroids’ where they felt they had to learn an ‘entirely new language’. Therefore, while online dating was seen to offer significant opportunities for Autistic GSM individuals, it also presented unique challenges.

### Theme 4: understanding and neurotype

Our final theme encompasses the notion of a romantic partner as someone who you could understand and who also understood you to a greater degree than others. This included feeling like their partner had consideration for their needs, not feeling judged, as well as a certain ease of communication. This is similarly related to the *Partner* as ‘designated human’ subtheme, but here the focus is on feeling understood as their Autistic self, as well as understanding their partner in return:

Nova: ‘he feels like the other half of my brain. He says the same thing about me. And so what I find is just really nice is that there’s no, I don’t have to put on an act. I don’t have to be a certain way. There’s no shame in being myself’.

Multiple participants noted that it was easier to achieve this high level of comfort, trust and understanding when their partner was also Autistic or neurodivergent. This was partly due to having a shared lived experience of being Autistic:

Renee: ‘Both of us are Autistic, so both of us have been through the wringer’.

Similarly, as understanding goes both ways, participants noted that it was also often easier to understand and relate to an Autistic partner; Penny noted that it was because of this that Autistic people tend to ‘just gravitate towards each other. Cause we get it’. This also extended to non-verbal understanding:

Masie: ‘I find that even with people who are Autistic and don’t have very many communications skills, I still find it quite easy to navigate interactions with them because I can kind of tell what they’re – how they’re kind of gonna work and I can relate to some of the stuff that’s difficult for them and then I can work around that’.

Along these lines, Marcy describes a situation in which their Autistic partner broke up with them over text, which was a really unpleasant experience for them. However, upon further consideration, she realized that this was due to her partner’s autism playing a role in how she felt comfortable communicating in such an emotionally charged situation. Marcy’s own lived experience of being Autistic helped them understand where their partner was coming from and realize that she hadn’t communicated the breakup over text to be hurtful.

However, participants noted that simply being Autistic did not mean that someone was going to be well-suited as their romantic partner. They brought up that two Autistic people could have conflicting needs, as well as the importance of compatibility across values, preferences and lifestyles. Furthermore, while a shared neurotype did not guarantee compatibility, a mismatch in neurotype did not preclude it:

Iris: ‘I’ve got this baseline of you know, pretty good relationship with a non-Autistic person. Actually despicable relationship with an Autistic person, and godly heavenly relationship with a non-Autistic person, so it’s not so possible to make generalizations’.

Participants also felt understood by their neurotypical partners, although they did note that it often involved ‘kind of having to explain things somewhat’. As understanding flows both ways, participants noted that understanding their neurotypical partners also took greater effort, with Nova equating the process to ‘learning each other’s languages’. Ultimately, multiple participants described having highly satisfying relationships with neurotypical partners in which both parties felt understood, safe and able to be themselves. In addition, it was also mentioned that Autistic people were happy to put effort into relationships as long as this effort is reciprocated:

Penny: ‘And I think for neurodivergent people, we’re already working pretty hard to fit into every other concept of society. So, we’re not afraid of working hard for relationships’.

Autistic people work incredibly hard to function in a world that was not designed with them in mind, and romantic relationships were also seen as requiring significant effort. As partnerships are a two-way street, being able to understand one’s partner, but also feeling reciprocal efforts in fostering understanding, was seen as fundamental.

## Discussion

The current study aimed to address a key knowledge gap by exploring the experiences and perceptions of romantic relationships in Autistic GSM groups. The findings show a recurring theme of rejecting prescriptive social norms – whether around what defines a partner, the role of sexual intimacy or even the structure of relationships captured by the theme – ‘It’s quite difficult to put Autistic relationships in a box’.

For many participants, physical intimacy or monogamy was not seen as requirements for a romantic relationship, and sex was not necessarily exclusive to romantic partners. This reflects previous work that Autistic participants viewed sex as less central to relationships compared to non-Autistic counterparts (Sala et al., 2024). Instead, sex was sometimes described as recreational or separate from love and romance, which resonates with our distinction between the subthemes of *Physical Intimacy* and *Partner as* ‘designated human’. In line with earlier findings, participants also described greater openness to non-monogamous arrangements, including polyamory (Sala et al., 2020).

When physical intimacy was removed from consideration, participants often did not elucidate tangible differences between friends and romantic partners. This was due to the fact that friends were also seen as highly valued people who understood them, which echoes past research (Gillespie-Smith et al., 2024). Partners were often referred to as their ‘best friend’, with many participants noting that their friendships were fulfilling in many of the same ways that a romantic relationship would be. Ultimately, the consensus was that the main factor that differentiates a partner from a friend is the label itself; rather than tangible differences, it comes down to a mutual decision to be romantic partners. Romantic partners were described as being highly integrated into their lives, with a high degree of emotional intimacy and mutual responsibility. This relates to past qualitative research in which Autistic adults described an ideal partner as someone they felt safe with that accepted, supported and worked to understand them (Cheak-Zamora et al., 2019; Sala et al., 2020).

Participants also reported that the shared experience of being Autistic, as well as a shared Autistic communication style tended to facilitate mutual understanding. They did also highlight, however, that simply being Autistic did not guarantee understanding and did not necessarily make someone compatible as a romantic partner. This is interesting when considering Milton’s (2012) double empathy theory, which predicts that paired neurotypes should communicate and understand each other better (Crompton et al., 2020a, 2020b; Sheppard et al., 2016). This would suggest that in romantic relationships, where shared understanding is vital, that communication would be more successful within neurotypes rather than across neurotypes. In line with this, Strunz et al., (2017) found that Autistic people whose partner was also Autistic were significantly more satisfied with their relationship. In contradiction to this, however, the current participants described highly successful relationships with non-Autistic partners in which they felt accepted and understood and that they were able to understand their partner in return. This suggests that double empathy theory may be better understood as a continuum of neurocultural learning rather than an Autistic-allistic binary, as has been previously suggested within the context of Autistic experiences and perceptions of friendship (Gillespie-Smith et al., 2024).

Achieving mutual understanding with a partner simply required effort on both parts to ‘learn each other’s language’. Previous research echoes this conceptualization of empathy, communication and understanding their partners’ perspective as ‘hard work’ that Autistic people must do, and that successful neurodiverse couples must do together (Sala et al., 2020; Smith et al., 2021). It is possible that our results differ from Strunz et al. (2017), who reported higher satisfaction among Autistic-Autistic couples, because our focus on Autistic GSMs highlights relationships that often involve negotiating additional layers of identity, boundaries and structure. Therefore, strong communication may be a prerequisite for navigating relationships, making it less about shared neurotype and more about shared willingness to engage in this work.

Our most central theme was *Challenging Social Norms*, which is in line with previous research suggesting that Autistic people are less likely to subscribe to societal norms compared to non-Autistic people (George & Stokes, 2018). For those who are both Autistic and part of a GSM, or ‘living under a double rainbow’, this rejection to conform is even more pronounced (Toft, 2024). Participants described difficulties with neurotypical social scripts around romance, which they also identified as creating vulnerabilities to abuse – findings consistent with past research (Barnett & Maticka-Tyndale, 2015; Cheak-Zamora et al., 2019; Kanfiszer et al., 2017; Sala et al., 2020).

There was emphasis on the need for relationships to be individualized to suit everyone involved. Explicit communication with one’s partner was used as a tool to understand each other’s needs and boundaries and to create a dynamic that worked specifically for them. This practice enabled participants to question common social norms surrounding what a relationship *should* look like, such as the expectation of monogamy, in favour of more flexible forms of ‘relationship anarchy’. Direct communication has previously been noted as a characteristic of Autistic interaction styles, as well as a common tool within polyamorous relationship structures, which Autistic people are more likely to endorse (Jackson-Perry, 2024; Sala et al., 2020). Similar dynamics have also been observed within kink and BDSM (i.e. Bondage and Discipline, Dominance and Submission, and Sadism and Masochism) contexts, where explicit boundary-setting and negotiated agreements are central (Delilah & Bertilsdotter Rosqvist, 2024). The authors additionally note that kink provides clear frameworks for communicating desires and limits, which can reduce uncertainty and allow partners to co-create intimacy in ways that are both structured and flexible. They also describe these spaces as potentially Autistic-friendly, offering multiple pathways for intimacy, such as non-verbal forms of communication, and reinforcing the value participants placed on clarity, negotiation and autonomy in their relationships.

Participants linked their ease in rejecting social norms to the intersection of their Autistic and GSM identities, which has been previously described by Toft (2024). Prior research shows that Autistic GSMs frequently report understanding gender differently and that being Autistic facilitated exploration of gender identity (Glackin et al., 2024; Kourti & MacLeod, 2019). For example, some Autistic participants described not identifying with gender categories at all, highlighting a tendency towards fluidity or even an absence of gender identity (Kourti & MacLeod, 2019). Autism has therefore been theorized as disrupting cisheteronormative models: because Autistic people are less bound to symbolic meanings and social scripts, they are free to ‘inhabit new identities of their own making’ (Kourti & MacLeod, 2019, p. 57).

Scholars have introduced concepts such as *autisexual* and *autigender* to capture how autism can act as a lens filtering experiences of sexuality and gender (Toft, 2023). These framings resist deficit-based interpretations, instead emphasizing how Autistic perspectives expose the fragility of socially constructed categories. In this sense, Walsh and Jackson-Perry (2021) argue that Autistic people may not necessarily be more likely to identify as GSM; rather, non-Autistic people may feel greater pressure to conform to normative categories, while Autistic people may be freer to express identities in more authentic ways. Jack (2012) also argues that gender and sexuality models should rather be reframed as neurotypical models, since they fail to account for neurodivergent experiences.

Identity development was also described as an interdependent process, with some individuals recognizing their Autistic identity first, which then facilitated recognition of queerness, and others identifying first as LGBT+ (i.e. Lesbian, Gay, Bisexual, Trans and other sexual identities and orientations) or kinky before later coming to understand themselves as Autistic (Bertilsdotter Rosqvist et al., 2024). These accounts illustrate how Autistic ways of being are both entwined with gender and sexuality and simultaneously destabilize rigid categories. Importantly, self-understanding of Autistic identity has also been shown to enhance romantic intimacy by fostering clearer communication and greater self-awareness in relationships (Sala et al., 2020; Smith et al., 2021). Taken together, these findings suggest that autism not only challenges deficit models of gender and sexuality but also provides a lens through which to rethink romantic relationships themselves – moving away from prescriptive norms and towards more authentic, self-defined forms of intimacy.

And finally, previous research highlighted that Autistic SM individuals find recognizing and conveying signals of romantic interest, as well as meeting potential partners challenging (Sala et al., 2020). One way around this is the use of online dating platforms, which have been identified as facilitators of Autistic relationships by aiding communication as well as enabling access to a greater pool of potential partners and similar others (Gillespie-Lynch et al., 2014; Gillespie-Smith et al., 2021; Roth & Gillis, 2015; Yew et al., 2021). The current participants described both perks and perils associated with online dating. Benefits included increased access to diverse people (thus increasing their dating pool), clear communication of expectations, and easy disclosure of GSM and neurodivergent identity. Difficulties, however, still remained around navigating romantic interactions online, particularly when it came to associated neurotypical and cis- and hetero-normative scripts.

### Limitations

Although the current study is the first to explore romantic relationships in Autistic GSM adults exclusively, our sample is still limited in that only individuals that were able to engage with online recruitment and interview processes were included. The results are also limited by the sample’s age range and lack of ethnic diversity, particularly as perceptions and experiences of romantic relationships have been shown to change and evolve both across cultures and over the lifespan (Goodwin, 2013; Sumter et al., 2013). Further research needs to include more diversity including different genders, sexualities, cultures and ethnicities.

More research is needed to explore intersectional experiences of being Autistic and being part of a GSM including around issues of identity, connectedness and mental health, since preliminary evidence suggests people with multiple minority identities are at increased risk of mental health problems (George & Stokes, 2018; Glackin et al., 2024; White et al., 2024). As mentioned earlier, healthy romantic relationships can provide support and protect against poor mental health outcomes; therefore, it is important we understand the Autistic GSM experiences and perceptions of these relationships. In line with this, the current participants also noted increased potential for abuse within relationships, similar to other important research highlighting higher levels of interpersonal violence and victimization experienced by Autistic people (Pearson et al., 2023, 2024). More research identifying avenues of support or informing better awareness and public campaigns could be beneficial for Autistic people.

## Conclusion

This study sought to explore how Autistic GSM adults experience and perceive romantic relationships. Overall, participants described their relationships as deeply individual, inherently valuable and shaped by unique preferences, mutual understanding and explicit communication. They also emphasized the fluidity between friendships and romantic partnerships, the important role of mutual understanding within relationships and the inherent value of rejecting prescriptive social norms in favour of individualized romantic relationship dynamics. Just as autism scholarship has challenged deficit models of gender and sexuality, our participants’ accounts invite a rethinking of romantic relationships themselves, outside of the cisheteronormative and neuronormative lens and towards self-defined, authentic forms of intimacy.

## Supplemental Material

sj-docx-1-aut-10.1177_13623613251407765 – Supplemental material for ‘It’s quite difficult to put Autistic relationships in a box’: A qualitative exploration of romantic relationships in gender and sexually diverse Autistic adults Supplemental material, sj-docx-1-aut-10.1177_13623613251407765 for ‘It’s quite difficult to put Autistic relationships in a box’: A qualitative exploration of romantic relationships in gender and sexually diverse Autistic adults by Tina Ciric, Luka CJ White, Claire Allison-Duncan, Ellen Maloney and Karri Gillespie-Smith in Autism

## Notes

1. This article capitalizes ‘A’ when referring to the Autistic identity – for more information, please see Pineo (2022).

2. Assigned female at birth.
