# Supplementary material for: ‘It’s quite difficult to put Autistic relationships in a box’: A qualitative exploration of romantic relationships in gender and sexually diverse Autistic adults
Source: Autism. 2025 Dec 29;30(3):682–94. doi: 10.1177/13623613251407765 (PMC12923615; doi:10.1177/13623613251407765)
Supplement: sj-docx-1-aut-10.1177_13623613251407765 – Supplemental material for ‘It’s quite difficult to put Autistic relationships in a box’: A qualitative exploration of romantic relationships in gender and sexually diverse Autistic adults [file sj-docx-1-aut-10.1177_13623613251407765.docx]

**Supplementary Information A_R1**

**Results in Full**

Four main themes were identified from the interview data:  1) “It’s Quite Difficult to Put Autistic Relationships Into a Box”, 2) Social Norms, 3) The Perks and Perils of Online Dating and 4) Understanding and Neurotype. It is important to note that the quotations chosen are representative of the sample and the themes.

**Theme 1: “It’s Quite Difficult to Put Autistic Relationships Into a Box”**

This theme encompasses the difficulty in categorizing autistic relationships due to the significance of traditional factors such as physical intimacy and exclusivity varying across individuals; as Eli noted “it’s quite difficult to put autistic relationships into a box”. Overall, there was a nuanced and interconnected understanding of relationships, with fluid boundaries between friendships and romantic partnerships.  This theme reflects the personalized nature of relationships and challenges traditional conceptualizations of romantic relationships. Four subthemes were identified: *Friendship, Physical Intimacy, Partner as “designated human”*, and *Labels*.

***Sub-theme 1.1: Friendship***

Overall, participants described friends as highly valued people, with whom there was shared understanding, trust, and feelings of safety. When asked to describe the distinction between friends and romantic partners, participants noted similar notions of connection across both types of relationship:

Masie: “I value my friends as much as I value my partners, and I feel like those things are very similar.”

Many participants referred to their partner as their “best friend”, suggesting friendship as a necessary foundation for a romantic relationship. However, the exact distinction between a romantic partner and a best friend remained generally unclear:

Masie: “So like when I’m close to a friend, I like build a very rich connection with them. And with partners, it’s similar thing I-I build a very sort of rich connection with them. I’d say I find the kind of distinction between like romantic and non-romantic connections to be kind of hard to understand.”

***Sub-theme 1.2: Physical Intimacy***

Participants expressed a range of perspectives on whether physical or sexual intimacy served as a boundary between friendships and romantic relationships. While some viewed sex as something that was reserved for a romantic partner, others felt it was more fluid or questioned its overall significance in defining relationship boundaries:

Sidney: “I used to believe that I had to be in a relationship with everyone that I slept with…Um, so I would kind of jump from relationship to relationship. Have a very monogamous mindset of a romantic relationship is someone that you’re having sex with. But now I generally sleep with most of my friends, so that isn’t what makes a relationship for me anymore.”

Participants also acknowledged common stereotypes that Autistic people were not interested in sexual intimacy, or that it was less important to them because they were autistic. This was widely seen as a harsh generalization:

Iris: “I do think it's damaging when people assume that we are absolutely all disinterested in sex or incapable of experiencing sexual attraction or any or any of this other stuff because it fundamentally is a misunderstanding and it downplays things that are actually very important to some of us, you know.”

Regardless of the perceived role of sexual intimacy as a distinguishing factor between friends and partners, participants generally acknowledged the presence of a “different kind of feeling” between friends and partners, which introduces our next subtheme.

***Sub-theme 1.3: Partner as “Designated Human”***

Participants generally agreed that a defining trait of romantic partners was the high level of companionship, understanding, and safety to be themselves that they felt in their presence:

Marina: “For me, it's all about feeling safe in another person's presence. Umm, into being able to be yourself and drop any masks.”

Participants also described the high degree to which they integrated their lives with a romantic partner, with much higher levels of “closeness” than one would have with other people.

Iris: “… if you’re very invested, you may end up thinking about planning either growing old together…So yeah, I don’t think people do that with their friends with benefits usually.”

Romantic relationships were also described as a give-and-take, with partners having more obligations and looking out for one another to a higher degree, which could be difficult for some people.

Darren: “Um... and also, I find... romantic relationships can be very... socially and emotionally demanding and difficult. In a way that friendships often aren’t, and they often come with difficult expectations.”

Participants also described a higher degree of emotional investment within romantic relationships, with partners having a large influence on their emotional state. This, paired with the high level of integration of lives between partners (such as the fusion of friend groups) meant that partners were very central figures in participants’ lives; Darren equated his partner to “gravity”, in that “things start to centre around this person”. This notion was also relevant within the context of breakups, as undoing this high level of integration was described as particularly distressing, with one participant describing a flare-up in suicidal thoughts following their breakup.

Amara: “So I think like losing both the person as a person, losing like the connections around that person, losing the routines that you’ve built up losing like, even just the stuff your physical environment changes. Like everything in your life is, it’s a big change...”

Ultimately, romantic partners were seen as uniquely central figures in participants’ lives, providing high levels of emotional intimacy and integration.

***Sub-theme 1.4: Labels***

Ultimately, while the defining characteristics of romantic relationships was considered to be fluid, participants generally echoed Darren’s statement that “if you basically get down to it it’s more about how you and the other person are defining the relationship rather than any actual listable differences”. For example, Sam made the point that the difference between getting coffee with a friend and getting coffee with a partner but calling it a “date” was simply a difference in how it was framed.

Darren: “Obviously you’ve got the kind of, I’m going to call them ‘socially-understood factors’, such as levels of physical intimacy… but I don’t think those inherently make something a relationship or a friendship in the sense where... we generally consider holding hands, or kissing, or having sex with someone to be something that happens in a romantic relationship but also happens in friendships? But not all friendships. And that’s kind of... that’s kind of decided by the people who are in those friendships.”

Sidney also described how with his last partner, their relationship was indistinguishable from when they called it a friendship to when they started framing it as a romantic relationship, stating that “…the only thing that made it a romantic relationship, made us partners, was having a mutual desire to have a label”. Ultimately, it depends on whether both parties agree to define their relationship as a romantic partnership – or, as Iris aptly describe, both must “say the magic words”.

**Theme 2: Social Norms**

The theme of Social Norms encompasses the complexities and challenges that GSM Autistic individuals face in navigating the societal expectations associated with romantic relationships. Participants described difficulties in understanding implicit rules, cues, and scripts related to relationships, with many questioning their inherent value altogether.  Building upon the previous theme of “It’s quite difficult to put autistic relationships into a box”, participants advocated for defining connections based on individual preferences rather than adhering to prescriptive norms. Overall, this theme underscores the potential for individuals to challenge and redefine social norms, paving the way for more authentic and individualized experiences. Three subthemes arose: *Romantic Norms, “Individual relationships should be just that, individual”,* and *Sexuality & Gender.*

***Sub-theme 2.1: Romantic Norms***

Participants often felt that they had a hard time understanding how they were supposed to navigate romantic relationships and the associated social scripts. In relation to dating in particular, participants felt like they were expected to “know the kind of patterns of interaction”, which Nova specifically describes as “…just kind of a nightmare, it’s where I have to try to present myself in a way that I don’t necessarily know how to do”.

Nova: “… I still don’t know what the dating rules are [laugh]… I don’t know when people, when people say in shows wait two days to text. That like I don’t know whether that’s real or whether that’s, I do not know. So to this day, I still don’t understand what a typical dating relationship’s like, um at least according to other people…And I think that’s a real crucial difference between Autistic and non-autistic people.... We don’t know those things off the bat… there is a lot stacked against you.”

Difficulties in interpreting these norms and cues were also brought up as a factor that increased vulnerability to abuse. This was due to a tendency to believe how others presented themselves and take romantic interactions at “face value”, unknowingly putting themselves at risk. Alana states that for this reason “It’s very easy [for Autistic people] to not see past the charms of a predatory narcissist and to not know any better.”:

Marina: “I think there’s just almost a bit of inherit danger of like, particularly being an AFAB^2^ Autistic person and sort of navigating romantic relationships. Because I have for sure found that, um, taking people at face value put me in really shit situations… because inherently being in romantic relationship is, is that level of vulnerability and it just puts you in a really shit place if you happen to have fallen for someone who doesn’t have very good intentions… I think just sort of the the aspect of it of, it’s thinking surely this person wants to initiate a relationship because they have a romantic interest in me, because why would I assume otherwise?”

Ultimately, participants describe differences in navigating and understanding the social norms, cues, and scripts surrounding romantic relationships. Most interestingly, participants’ difficulty grasping social cues results in two very different approaches to dating and romantic relationships, which Sam summarizes:

Sam: “I think maybe Autistic people are. Well, some Autistic people can be less concerned with like social rules. And so like, maybe sure you’d like for social rule says you probably shouldn’t like, get together with someone after a couple of dates. But like, why should you care? But other Autistic people are very, very invested in social rules because they struggle to understand, like the flexibility in them. And so they tend to take them quite rigidly.”

Both of these approaches are illustrated by Renee and Masie; Renee states that they feel like “when you are neurotypical, those really rigid ideas that have been implanted through media don’t exist to the degree that they do in myself… it takes work to get out of that mentality”. This is echoed by Masie’s who said that in the past she “…got very sucked into, like, trying to be, like normal…” until realizing that she didn’t prescribe to these norms:

Masie: “You kind of can’t participate in the sort of neurotypical like social landscape in the same way that neurotypical people can. You also aren’t invested in it in the same way.”

Ultimately, it seems like the disorientation with prescriptive romantic social norms could facilitate their rejection, and allow for individualization of what romantic relationships are for each participant; as noted by Sam, “you just make it up and then that’s fine”.

***Sub-theme 2.2: “Individual Relationships Should Be Just That, Individual”***

It was widely agreed that ultimately it is up to each couple to decide what their rules and criteria are going to be for their individual relationship, regardless of societal norms. This subtheme is connected to the previous subtheme of *Romantic Norms* as differences understanding social norms was suggested to make it easier to pave their own way in terms of what a relationship looked like, which was echoed by multiple participants:

Penny: “…a lot of social expectations around relationships are very prescriptive. Like I can remember being told by my friends that it was outrageous if me to not be able to define what cheating was without talking to my partner. And I was like, well, how do I? Surely cheating depends on what you and your partner agree is cheating. They’re like, no, it’s kissing anyone else. I’m like, well for some people. Yeah, so and that was when I was in my 20’s. So, I was already of that mindset of each individual relationship should be just that, individual.”

Something that multiple participants spoke on was the norm of monogamy, with Zia noting that their past relationships had ultimately failed because “they were both convinced they could talk me into having marriage and kids. It’s like, yeah, it’s not happening”. Participants were very vocal about not compromising on the things that were important to them in a relationship, and displayed strong boundaries:

Sidney: “Yeah, I-I do always make sure that before I get with someone they know fully, like my boundaries… And what I want and it’s up to them to know if they can handle that or not.”

Marcy: “But yeah, my friend asked me… the girl who got me on Tinder, she’s like, so have you learned your lesson? How are you not gonna tell people that you want monogamy after the second date? I was like, no, I haven’t learned my lesson. I am very happy that I said what I said because neither of us wasted each other’s time. We can now move on. We can be friends. And if I’m in a similar situation to that again, I don’t want to see where it goes. I want to know that this is going to go in a place where both of us are are happy with it going. But I thought it was really interesting because obviously like she would have done things very, very differently. And again neurotypical thinking… But I just said, like, if there’s a possibility, this is what I would like. And I’m really not upset that I said that… I’m actually quite proud of it.”

This also appeared in relation to participants deciding for themselves what a breakup would look like within the context of their relationship:

Darren: “I also think... there’s a kind of feeling that ‘inherently this is a break up and therefore this is what we need to do’, if that makes sense? Like we need to stop talking, we need to have a massive flight, it’s gotta end in tears. It’s like, we really don’t have to end like this. Whereas like, I found that there was a lot more like... ‘ok we can actually decide what this is going to look like’…There’s a lot more ‘we’re getting to look at it and pick and choose’ rather than, like, ‘this is a script that we need to follow for breaking up’… And it meant that we’re still friends, and we get along great. But, it also means that a lot of people I meet find the fact that we did stay friends, and that I’m friends with his new partner, and that we get on, and all of that, some people I find find it a bit... unusual? Strange? They often have questions, and I think that’s because we got to pick it rather than following the script on it.”

At the end of the day, participants agreed that there was a much broader spectrum of what romantic relationships could look like than is generally portrayed. Rather than focusing on what relationships “should” look like, participants emphasized the value of individualization based on the values and preferences of each person.

Iris: “my current primary partner is somebody who I am romantically in love with and he does not romantically love me back, but we are, for want of a better word, friends with benefits and adventure companions. Umm, and even though I’m well aware that there’s a mismatch in our feelings towards one another, I actually don’t particularly mind because he’s never failed to be an absolutely perfect adventure companion and absolutely incredible lover”

This reflects the flexibility and agency participants embraced in defining relationships on their own terms, and how in the end all that matters is that both parties are satisfied with the state of their relationship.

***Sub-theme 2.3: Sexuality & Gender***

While this analysis has previously discussed autistic traits potentially facilitating the rejection of social norms, participants also suggested that their identity as a gender and sexual minority could play a further role in this:

Sidney: “…the trans/Autistic people I’ve dated just generally have a disillusionment with the cis/hetero institutions of marriage and children and stuff, so I don’t like know if it’s because we’re Autistic or if it’s because we’re trans.”

Darren: “I think this is possibly because I'm gay, um... a lot of things that some people think are exclusively to romantic relationships I don’t see as inherently being like that, so long as everyone is, like, on the same page about it, if that makes sense?”

Multiple participants also mentioned the high amount of overlap they had noticed between the autism and polyamory communities, as well as the high amounts of gender and sexual diversity within the Autistic community. In relation to the kink and polyamory communities in particular, the direct communication style and freedom granted by their inherent rejection of normative relationship frameworks was seen as appealing and beneficial:

Penny: “I think because of the freedom I have to communicate that…exists within polyamory, reduction of expectations…Has definitely benefited me, especially where I am now because...[I] probably wouldn’t be dating otherwise.”

Masie: “You know, the kink scene is great for Autistic people. You've maybe encountered this already in your research because there's lots of explicit rules about how you're supposed to interact with people. And that's the space which is, like, very accessible.”

Therefore, the intersection of identifying as Autistic, a GM, and an SM, could foster the rejection of traditional relationship expectations, allowing for greater diversity in relational dynamics.

**Theme 3: The Perks and Perils of Online Dating**

In general, online dating was acknowledged as an appealing alternative to traditional avenues of meeting a partner, allowing participants to do so on their own terms:

Penny: “Absolute nightmare, because where do all the non-neurodivergent people meet? Clubs! Where do I hate with the passion of 1000 Suns? Clubs!”

Dating apps were described as “removing a lot of the obstacles”, “good for filtering people”, granting access to a “more diverse range of people”, and “speeding up the process” of finding a partner. This was seen as particularly useful for GSMs in particular when it came to finding potential partners or communicating their desired relationship structures (i.e. non-monogamy):

Jonathan: “…another step, quite a massive step, when you’re gay, which is figuring out whether they’re queer in the first place. Umm... so, uhh.. I think I think that Tinder and apps like that, they really speed along the process because you know they’re on there to date, in fact you know that everyone you match with is into you, so it’s even better”

However, participants also noted certain difficulties associated with online dating, specifically surrounding social norms, cues, and scripts. Dating in a virtual space was described as the “neurotypical world on steroids” where they felt they had to learn an “entirely new language”. Participants particularly highlighted the unspoken rules around interaction, complicated scripts around how you’re meant to show interest, and equated the entire process to feeling like they were “playing a game”. This ultimately drove many of them away from using online dating platforms. Therefore, while online dating was seen to offer significant opportunities for Autistic GSM individuals, it also presented significant barriers and challenges.

**Theme 4: Understanding & Neurotype**

  Our final theme encompasses the notion of a romantic partner as someone who you could understand and who also understood you to a greater degree than others. This included feeling like their partner had consideration for their needs, not feeling judged, as well as a certain ease of communication. This is similarly related to the *Partner as “designated human”* subtheme, but here the focus is on feeling understood as their Autistic self, as well as understanding their partner in return. Participants described feeling able to relax and be their Autistic selves around their partner, particularly in terms of not having to mask:

Nova: “…he feels like the other half of my brain. He says the same thing about me. And so what I find is just really nice is that there’s no, I don’t have to put on an act. I don’t have to be a certain way. There’s no shame in being myself.”

Multiple participants noted that it was easier to achieve this high level of comfort, trust, and understanding when their partner was also Autistic or neurodivergent. This was partly due to having a shared lived experience of being Autistic:

Marina: “But it does really help in the way that like in the sense that he knows and because his experience that he knows what it’s like to live in a world that’s not built for you and how it ties to try and like accommodate your own home, for to yourself, and then trying to cope with what you can change on the outside. Umm, so the there definitely is a different level of understanding I think.”

Renee: “Both of us are Autistic, so both of us have been through the wringer.”

Similarly, as understanding goes both ways, participants noted that it was also often easier to understand and relate to an Autistic partner; Penny noted that it was because of this that Autistic people tend to “just gravitate towards each other. Cause we get it”. This also extended to non-verbal understanding:

Masie: “I find that even with people who are Autistic and don’t have very many communications skills, I still find it quite easy to navigate interactions with them because I can kind of tell what they’re—how they’re kind of gonna work and I can relate to some of the stuff that’s difficult for them and then I can, you know, and then I can work around that.”

Along these lines, Marcy describes a situation in which their Autistic partner broke up with them over text, which was a really unpleasant experience for them. However, upon further consideration, she realised that this was due to her partner’s autism playing a role in how she felt comfortable communicating in such an emotionally-charged situation. Marcy’s own lived experience of being Autistic helped them understand where their partner was coming from, and realize that she hadn’t communicated the breakup over text to be hurtful.

Participants also agreed that along with a shared experience of being Autistic, similarities in communication style with Autistic partners was also a big facilitator of understanding as they could “speak the same silly”. This communication style was often described as “direct” and “blunt”.

Masie: “I find it’s a lot easier for me to connect with and communicate with other Autistic people. I find a lot more joy and a lot more understanding and a lot more comfort than in like my-like uh, for relationships I guess.”

Darren also describes his past relationship with an Autistic partner as having “…very direct, very blunt, very clear... communication, which meant that a lot of the kind of stress of trying to figure out where you stood with someone, trying to figure out what the other person was thinking, wasn’t something I had to do”. He also goes on to describe the monthly “State of the Union” meetings they would have:

Darren: “…we would just sit down and go ‘right, let’s talk about it’. You know, how do we feel about the number of dates we’re going on? How do we feel about how much we’re talking? How do we feel about how it’s cutting into other aspects of our lives? … Like a very simple, almost like check-in list to discuss it, rather than having to have something where, in other ones where it’s reached a kind of boiling crisis point before problems have come up, and I found that SO much easier to work with, and so much easier to kind of adapt to.”

However, participants noted that simply being Autistic did not mean automatically that someone was going to be well-suited to being their romantic partner. They brought up that two Autistic people could have conflicting needs, as well as the importance of compatibility across values, preferences, and lifestyles. Furthermore, while a shared neurotype did not guarantee compatibility, a mismatch in neurotype did not preclude it:

Iris: “… I’ve got this baseline of you know, pretty good relationship with a non- Autistic person. Actually despicable relationship with an Autistic person, and godly heavenly relationship with a non-Autistic person, so it’s not so possible to make generalizations.”

Participants also felt understood by their neurotypical partners, although they did note that it often involved “…kind of having to explain things somewhat”. As understanding flows both ways, participants noted that understanding their neurotypical partners also took greater effort, with Nova equating the process to “learning each other’s languages”. Ultimately, multiple participants described having highly satisfying relationships with neurotypical partners in which both parties felt understood, safe, and able to be themselves. Masie noted that all it takes is just that extra bit of effort to understand where your partner is coming from, which is not a lot to ask for seeing as Autistic people spend so much time learning to fit in to a neurotypical world:

Masie: “…it’s not like neurotypical people cannot understand Autistic people. It’s like you just gotta put a little bit of time in. Put, like, 1/10th of the amount of work into understanding how we work that we put into understanding how you work.”

Penny: “And I think for neurodivergent people, we're already working pretty hard to fit into every other concept of society. So, we're not afraid of working hard for relationships.”

Autistic people work incredibly hard to function in a world that was not designed with them in mind, and romantic relationships were also seen as requiring significant effort. As partnerships are a two-way street, being able to understand one’s partner, but also feeling reciprocal efforts in fostering understanding, was seen as fundamental.
